# Immunostimulation Signaling via Toll-like Receptor 2 Activation: A Molecular Mechanism of *Lactococcus lactis* OTG1204 In Vitro and In Vivo

**DOI:** 10.3390/nu16213629

**Published:** 2024-10-25

**Authors:** Hyeon-A Song, Seo-Yun Jang, Min-Ji Park, Seung Wook Kim, Choon Gil Kang, Joo Hyun Lee, Hye-Jin Kim, Jiheon Kim, Jong Kil Lee, Kyung-Sook Chung, Kyung-Tae Lee

**Affiliations:** 1 Department of Pharmaceutical Biochemistry, College of Pharmacy, Kyung Hee University, Seoul 02447, Republic of Korea; sha3076@naver.com (H.-A.S.); tjdbs2357@naver.com (S.-Y.J.); mjpark957@naver.com (M.-J.P.); adella76@hanmail.net (K.-S.C.); 2 Department of Fundamental Pharmaceutical Science, Graduate School, Kyung Hee University, Seoul 02447, Republic of Korea; jklee3984@khu.ac.kr; 3 Ottogi Research Center, Anyang 14060, Republic of Korea; kimsw@ottogi.co.kr (S.W.K.); gilkang@ottogi.co.kr (C.G.K.); joohyun_lee@ottogi.co.kr (J.H.L.); khj0284@ottogi.co.kr (H.-J.K.); kjh0085@ottogi.co.kr (J.K.)

**Keywords:** probiotics, *Lactococcus lactis* OTG1204, immune, macrophages, cyclophosphamide, gut microbiota

## Abstract

Introduction: The immune system’s defense against pathogens involves innate and adaptive responses, crucial in maintaining overall health. Immunosuppressed states render individuals more susceptible to potential diseases, indicating the need for effective strategies to bolster immune functions. Objectives: Although the immunostimulatory effects of various probiotics have been studied, the specific effects and molecular mechanisms of *Lactococcus lactis* OTG1204 (OTG1204) remain unknown. In this study, the aim was to investigate the molecular mechanisms of OTG1204 in RAW 264.7 macrophages, the key effector cells of the innate immune system involved in host defense and inflammatory responses. Additionally, in this study, the effects of OTG1204 on cyclophosphamide (CTX)-induced immunosuppression states were investigated, thereby demonstrating its potential as an immune stimulant. Methods: To assess the macrophage activation ability and underlying mechanisms of OTG1204, RAW 264.7 cells were utilized with transfection, enzyme-linked immunosorbent assay, and quantitative real-time PCR analyses. Furthermore, to evaluate the immunostimulatory effects under immunosuppressed conditions, CTX-induced immunosuppression mice model was employed, and analyses were performed using hematoxylin and eosin staining, flow cytometry, and microbiota examination. Results: OTG1204 activated RAW 264.7 macrophages, leading to increased production of nitric oxide, prostaglandin E2, and cytokines. This immune activation was mediated through the upregulation of toll-like receptor 2, which subsequently activated the nuclear factor-κB (NF-kB) and mitogen-activated protein kinase (MAPK)/activator protein 1 (AP-1) pathways, thereby stimulating the immune response. In CTX-treated mice, OTG1204 recovered body weight, spleen, and mesenteric lymph node indices, and natural killer cell activity. It re-established populations of innate and adaptive immune cells and activated T cells to secrete cytokines. We also examined the gut barrier integrity and microbiota composition to assess OTG1204’s impact on intestinal health, as these factors play a significant role in immune enhancement. OTG1204 enhanced gut barrier integrity by upregulating mucin 2 and tight junction proteins and modulated the gut microbiota by restoring the *Firmicutes*/*Bacteroidetes* balance and reducing the abundance of *Actinobacteria* and *Tenericutes*. Conclusion: These results suggest that OTG1204 may serve as an effective probiotic for immune enhancement and gut health management by targeting the NF-κB and MAPK/AP-1 pathways, with minimal side effects.

## 1. Introduction

The immune response involves various immune cells and proteins that orchestrate responses to defend against non-self-substances [1]. When an antigen attacks the host, macrophages initiate the immune response by recognizing pathogen-associated molecular patterns on invading pathogens through pattern recognition receptors like toll-like receptor 2 (TLR2) [2]. Activation of TLR2 triggers the downstream signaling pathways, including nuclear factor-κB (NF-κB) and mitogen-activated protein kinase (MAPK), which induce the expression of immune mediators that regulate the survival, activation, and differentiation of immune cells [3]. TLR2 not only initiates the immune response but also shapes the subsequent immune signaling cascades. As the early immune response begins, macrophages release pro-inflammatory cytokines, including interleukin-1β (IL-1β), tumor necrosis factor-α (TNF-α), and interleukin-6 (IL-6). These cytokines recruit additional immune cells, such as neutrophils and dendritic cells, while enabling macrophages to process and present antigens to T cells, bridging the innate and adaptive immune systems [4]. Along with macrophages, natural killer (NK) cells contribute to the early immune response by detecting and eliminating infected or abnormal cells, providing an immediate defense before adaptive immunity is fully activated [5]. Adaptive immunity includes B cell-producing antibodies and T cells that facilitate pathogen elimination and memory, thus playing a precise and effective role in pathogen defense [6].

With the increasing prevalence of autoimmune diseases and immune dysregulation, the development of immunostimulatory agents to modify immunity and regulate immunological disorders has become increasingly important [7]. Immunosuppression, often induced by chemotherapy or other medical treatments, weakens the immune system, making individuals more susceptible to infections [8]. To mimic such conditions, cyclophosphamide (CTX) is commonly used as an immunosuppressive agent in experimental models. CTX reduces immune cell numbers and impairs their function, providing a model to evaluate the potential of immunostimulatory agents like probiotics in restoring immune function under compromised conditions [9]. In this regard, probiotics have gained attention for their potential as immunostimulatory agents. They enhance non-specific host defenses against microbial pathogens and downregulate hypersensitivity reactions by modulating the immune system, influencing cytokine profiles, and improving intestinal barrier function [10]. Probiotics also exert beneficial effects on the intestine by regulating intestinal permeability and gut microbiota composition [11]. The complex relationship between gut immune cells, microbiota, and mucosal immunity plays a pivotal role in defending against pathogens [12]. Tight junction proteins and mucin 2 (MUC2) maintain the integrity of the intestinal barrier and control the selective permeability of the intestinal lining, thereby aiding in immune defense and promoting overall gut health [13]. Moreover, the gut microbiota is instrumental in balancing the presence of harmful and beneficial bacteria that are crucial for gut homeostasis. This equilibrium also supports the development of both the innate and adaptive immune systems, demonstrating the multifaceted role of probiotics in enhancing immune function and maintaining health [14]. Both tight junction integrity and microbiota composition are essential components in immune enhancement, as they help maintain a stable and responsive immune system.

Among the various probiotic strains, *Lactococcus lactis* has been extensively studied for its immunostimulatory properties. *Lactococcus lactis*, commonly found in fermented foods such as cheese and milk, can adhere to the intestinal mucosa, supporting its potential for beneficial interactions within the gut [15]. *Lactococcus lactis* OTG1204 (OTG1204), isolated from kimchi, a traditional Korean dish, has not been previously studied for its immunostimulatory effects. Other strains, such as *L. lactis* GCWB1176, increase the cytokine levels and NK cell activity [16]. In addition, *L. lactis* subsp. *cremoris* RPG-HL-0136 supports the growth of a beneficial microbiome by enhancing intestinal immune function [17]. Given the recognized health benefits of *L. lactis*, we hypothesized that *L. lactis* OTG1204 may also be advantageous for immune stimulation. Therefore, we investigated the immunostimulatory effects of OTG1204 and its molecular mechanism in RAW 264.7 macrophages and a CTX-induced immunosuppression mice model. This study was performed to gain insights into how OTG1204 potentially enhances immune responses, contributing to its application in improving gut health and immunity.

## 2. Materials and Methods

### 2.1. Materials

Anti-mTLR2-IgG (#mabg-mtlr2), and Mouse Control IgG2a (#mabg2a-ctrlm) antibodies were obtained from InvivoGen (San Diego, CA, USA). Polymyxin B sulfate, lipopolysaccharide (LPS) from *Escherichia coli*, and cyclophosphamide (CTX) were purchased from Sigma-Aldrich (St. Louis, MO, USA). 

### 2.2. Preparation of OTG1204

OTG1204 in a lyophilized powder form was provided by the Ottogi Research Center (Anyang, Republic of Korea). The lyophilized powder (3.0 × 10^11^ CFU/g lyophilized powder) was then stored at 4 °C until further use. OTG1204 was deposited in the Korean Collection for Type Cultures (KCTCs) under accession number KCTC 15870BP.

### 2.3. In Vitro OTG1204 Treatment

RAW 264.7 mouse macrophage was obtained from ATCC (Manassas, VA, USA). RAW 264.7 cell culture was conducted following ATCC’s recommended protocols. RAW 264.7 cells were seeded at a density of 2 × 10^5^ cells/mL in plates and incubated overnight. The seeded cells were treated with OTG1204 (0.5 × 10^6^, 1 × 10^6^, or 2 × 10^6^ cfu/mL) or LPS (5 ng/mL, positive control).

### 2.4. Cell Viability

The 3-(4,5-dimethylthiazol-2-yl)-2,5-diphenyltetrazolium bromide (MTT) assay was conducted for the cell viability assessment. After treatment with OTG1204 for 24 h, MTT solution was added to cells for 3 h at 37 °C. The violet formazans of cells were dissolved using dimethyl sulfoxide and the absorbance was measured at 540 nm.

### 2.5. Measurement of Nitric Oxide (NO)

The concentration of NO in the culture supernatants was estimated using the Griess reaction, and its standard curves were prepared using NaNO_2_. After treatment with OTG1204 for 24 h, the level of nitrite, as an indirect indicator of NO production, was detected by measuring the absorbance at 540 nm.

### 2.6. Measurement of Cytokine and Prostaglandin E_2_ (PGE_2_)

Cytokine production levels in the culture supernatants of RAW 264.7 cells (IL-1β, IL-6, TNF-α) or ConA-stimulated T cells from splenocytes of CTX-induced immunosuppressed mice (TNF-α, IFN-γ, IL-12, IL-4, IL-6) were measured by enzyme-linked immunosorbent assay (ELISA) kits (R&D Systems, Minneapolis, MN, USA), following the manufacturer’s instructions. After treatment with OTG1204 for 24 h, the PGE_2_ production in the culture supernatants from RAW 264.7 cells was measured using a PGE_2_ ELISA kit (Enzo Life Sciences, Farmingdale, NY, USA). 

### 2.7. Quantitative Real-Time PCR (qRT-PCR)

The total RNA of cell and animal tissue was extracted by Easy Blue^®^ kits (Intron Biotechnology, Seoul, Republic of Korea). The quantified mRNA (500 ng) was converted to cDNA with random oligonucleotide primers (Promega, Madison, WI, USA) and TOP script^TM^ RT DryMIX (Enzynomics, Daejeon, Republic of Korea). The target genes were amplified using the QuantStudio 1 (Thermo Fisher Scientific, Waltham, MA, USA) with various primers (Table 1). 

### 2.8. Luciferase Reporter Assay Using Cellular Transfection

RAW 264.7 macrophages were co-transfected with the luciferase vector (pNF-κB-Luc or pAP-1-Luc, Clontech, Shiga, Japan) and the control vector phRL-TK plasmid (Promega, Madison, WI, USA) using Nucloefector^TM^ 2D device (Lonza, Valais, Switzerland) under the manufacturer’s instructions. Transfected cells with pNF-κB-Luc or pAP-1-Luc were treated by OTG1204 for 6 h or 24 h, respectively. Luciferase activity was estimated using the Dual-Luciferase^®^ Reporter Assay System (Promega, WI, USA) on a Spectramax L Microplate Luminometer (Molecular Devices, Toronto, ON, Canada).

### 2.9. Western Blot Analysis

Whole proteins of cells and colon tissues were extracted using the PRO-PREP™ protein lysis buffer (Intron Biotechnology, Seoul, Republic of Korea). Cellular nuclear or cytosol fraction was prepared as described previous report [18]. Quantified proteins and fractions (20 μg) were separated by sodium dodecyl sulfate-polyacrylamide gel electrophoresis and immunoblotted with target protein-specific antibodies (Supplementary Table S3) [19].

### 2.10. Ethics Statement

All experimental procedures complied with the Animal Care and Use guidelines approved by Kyung Hee University (KHSASP-23-039).

### 2.11. Cyclophosphamide-Induced Immunosuppression in Mice

**Figure 1:**
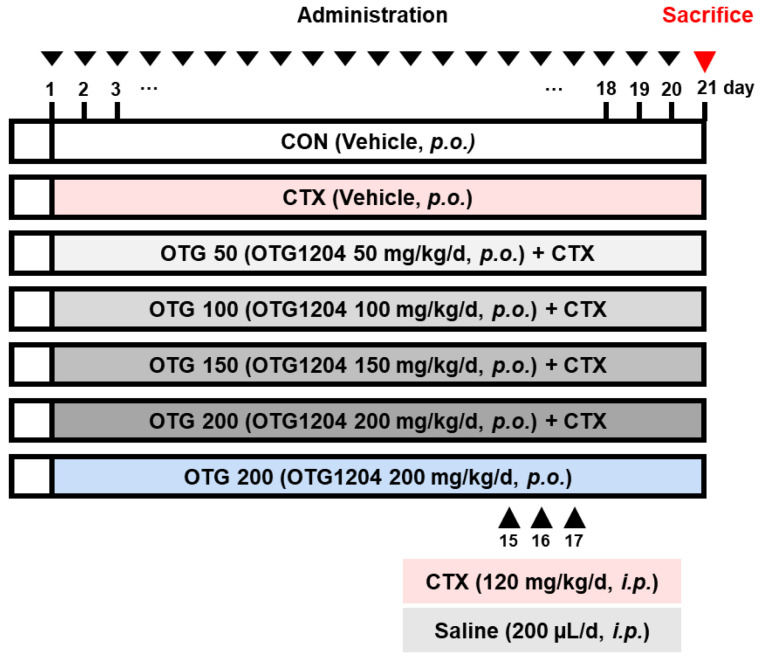
Scheme of the in vivo study.

To establish an immunosuppression animal model, we used a C57BL/6 mice strain because they are justified an immune response to CTX treatment [20,21]. C57BL/6 (male, 6 weeks) was obtained from Orient Bio Inc. (Seongnam-si, Republic of Korea). They were housed under controlled conditions at 22 ± 1 °C and 50 ± 10% humidity, with a 12 h light/12 h dark cycle and free access to food and water at the Kyung Hee University animal facility. The research team monitored the animals every day. The strategy used to minimize potential confounders involved administering treatments to each group and within each group in the same order and at the same time each day. Mice were randomly divided into seven groups, each consisting of seven mice; normal control group (CON group), CTX-treated group (CTX group), OTG1204 50 mg/kg/day + CTX-treated group (OTG 50 + CTX), OTG1204 100 mg/kg/day + CTX-treated group (OTG 100 + CTX), OTG1204 150 mg/kg/day + CTX-treated group (OTG 150 + CTX), OTG1204 200 mg/kg/day + CTX-treated group (OTG 200 + CTX), and OTG1204 200 mg/kg/day group (OTG 200) (Figure 1). During the experiment, the CON and CTX groups were gavaged with the vehicle 200 μL/day, while the other five groups were orally gavaged with OTG1204 (50, 100, 150, or 200 mg/kg/day) for 20 days. On the 15th, 16th, and 17th days, the CON and OTG1204 200 mg/kg/day single-treated groups were intraperitoneally injected (i.p.) with saline, while all other groups were treated with CTX (120 mg/kg, i.p.) once a day for 3 days. On the 21st day, collected feces were immediately frozen in nitrogen liquid and the blood, spleens, mesenteric lymph nodes (MLNs), and large intestines were excised for further experiments.

### 2.12. Spleen and MLN Index

The spleen index and MLN index were calculated using the following formulas:

Spleen Index (mg/kg) = Spleen weight (mg)/Body weight (kg)

MLN Index (mg/kg) = MLN weight (mg)/Body weight (kg)

### 2.13. Splenocyte Isolation

The spleen was dissected and placed in a Roswell Park Memorial Institute (RPMI) medium involving 10% fetal bovine serum and 1% penicillin and streptomycin (Hyclone, Logan, UT, USA). The spleen was pressed and sliced using two slide glasses and splenocytes were filtered through a cell strainer (70 µm pore). Isolated splenocytes were centrifuged at 2400 rpm for 10 min and medium supernatants were discarded. The splenocytes were reacted with the eBioscience^TM^ X RBC Lysis Buffer (Invitrogen, Carlsbad, CA, USA) for 5 min and then centrifuged at 2200 rpm for 5 min. The splenocytes were resuspended in RPMI-1640. For cytokine analysis, the isolated splenocytes were stimulated with concanavalin A (ConA) treatment (5 μg/mL).

### 2.14. NK Cell Activity

The NK cell activity was calculated by assessing the cytotoxicity of NK cells (effector cells) to YAC-1 cells (target cells) with an LDH assay kit (Sigma-Aldrich, St. Louis, MO, USA). The splenocytes were seeded in 96-well plates at 1 × 10^6^ cells/mL, and after 1 h incubation, mouse interleukin 2 (mIL-2) (50 ng/mL) was treated for 48 h. Then, YAC-1 cells were seeded 5 × 10^6^ cells/mL onto the splenocyte. In the high control wells, YAC-1 cells were seeded 5 × 10^6^ cells/mL and Triton X was added, while in the low control wells, YAC-1 cells were seeded 5 × 10^6^ cells/mL and RPMI medium was added for 4 h. After the 96-well plates were centrifuged at 2400 rpm for 5 min, supernatants from each well were reacted with LDH substrate mixture. The absorbance of each well was measured at 490 nm. The percentage of NK cell cytotoxicity was calculated using the following formula:

$$\mathrm{NK}\;\mathrm{cell}\;\mathrm{cytotoxicity}\;(\%)=\frac{experimental\;release-spontaneous\;release}{maximum\;release-spontaneous\;release}\times 100$$

### 2.15. Composition Analysis of Immune Cells in the Isolated Splenocytes

After splenocyte isolation, splenocytes were incubated in cell-staining buffer with TruStain FcX (anti-mouse CD16/32) for 10 min. Then, the splenocytes were reacted for 20 min in the dark with fluorescent antibodies (BioLegend Inc, San Diego, CA, USA), as follows: PerCP anti-mouse CD3, CD11c and Ly6G, PE anti-mouse CD4, NK1.1, CD11b, MHCII and Ly6c, FITC anti-mouse CD19 and F4/80, and APC anti-mouse IFN-γ, IL-4, and B220. Fluorescent antibody-stained splenocytes were analyzed by a flow cytometer (Beckman Coulter^®^, Brea, CA, USA). 

### 2.16. Histological Analysis

The colonic tissues were fixed in 4% paraformaldehyde overnight, and the tissue was embedded in paraffin. The colonic tissues in paraffin blocks were cut and then stained with hematoxylin and eosin (H&E) for the analysis of tight junction changes and crypt length.

### 2.17. Gut Microbiota Analysis

Total genomic DNA (gDNA) was extracted from stools using the QIAamp^®^ Fast DNA Stool Mini Kit (Qiagen, Hilden, Germany), according to the manufacturer’s protocol. The V3–V4 region of the bacterial 16S ribosomal RNA gene was amplified by PCR and the amplified products were purified with a magnetic bead method. Equal concentrations of all purified products were combined to construct a genomic library, which was quantified to 2 nM using KAPA Library Quantification Kit Illumina^®^ platforms (Roche, Basel, Switzerland), and the microbiome taxonomic profiling was performed on the Illumina iSeq 100 sequencing system (Illumina, San Diego, CA, USA). Beta diversity was analyzed using both unweighted UniFrac distance and weighted UniFrac distance through principal coordinate analysis (PCoA) and PCoA was visualized using the Plotly platform (https://chart-studio.plotly.com, accessed on 5 November 2023).

### 2.18. Statistical Analysis

Data are expressed as the mean ± SD for in vitro experiments and mean ± SEM for in vivo experiments. Statistical significance was evaluated using one-way analysis of variance (ANOVA) and Dunnett’s post hoc test. A *p*-value of less than 0.05 was considered to indicate statistical significance. The correlation between gut microbiota and immune factors, including immune cells and cytokines, or tight junction proteins was analyzed using the Pearson correlation coefficient.

## 3. Results

### 3.1. OTG1204 Stimulates the Production and Expression of Immunostimulatory Mediators in RAW 264.7 Macrophages

**Figure 2:**
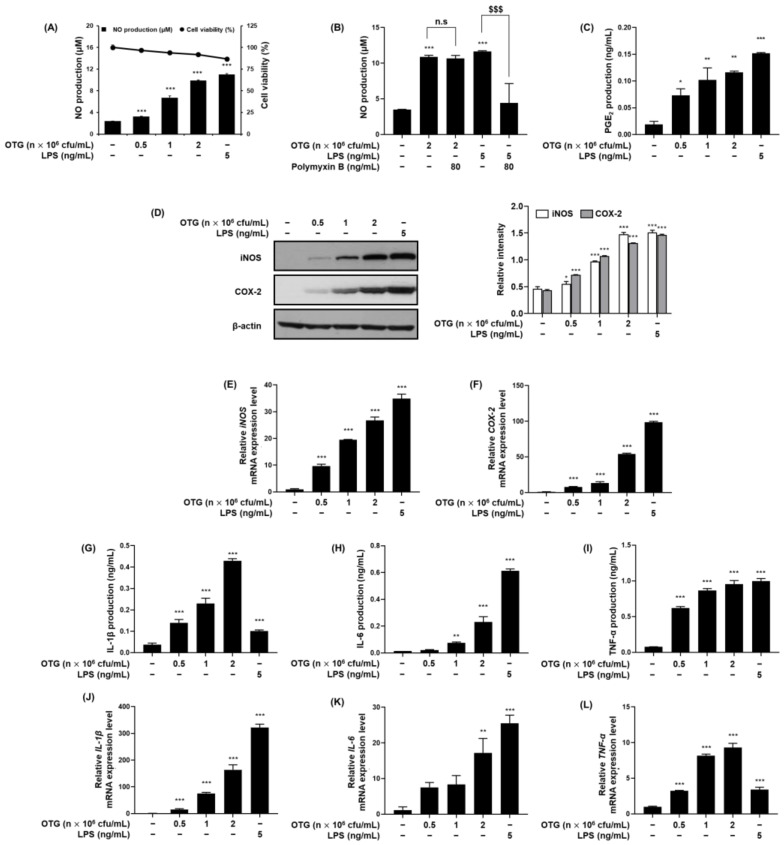
The immunostimulatory activities of OTG1204 in RAW264.7 macrophages. (**A**,**B**) The cell viability of OTG1204 was analyzed by MTT and NO production was detected by Griess reaction. LPS (5 ng/mL) was used as the positive control. For endotoxin contamination tests, cells were pretreated with or without Polymyxin B (80 ng/mL) for 1 h and then treated with OTG1204 for 24 h. (**C**) The PGE_2_ production was measured by ELISA kits. (**D**) The protein expressions of iNOS and COX-2 were detected by Western blot analysis. β-actin was used as the internal control. (**E**,**F**) The mRNA expressions of iNOS and COX-2 were detected by qRT-PCR analysis. (**G**–**I**) The production and (**J**–**L**) mRNA expression of IL-1β, IL-6, and TNF-α were measured by ELISA kits and qRT-PCR, respectively. Data are expressed as means ± SD. * *p* < 0.05, ** *p* < 0.01, *** *p* < 0.001 vs. Control. ^$$$^ *p* < 0.001 vs. LPS treatment. n.s.: not significant.

We screened twenty lactic acid bacteria (LAB) strains for immunostimulatory activity by evaluating the cytotoxicity and NO production in RAW 264.7 macrophages. As shown in Supplementary Table S1, *Lactiplantibacillus plantarum* OTG1039, *Limosilactobacillus fermentum* OTG1050, and *Lactococcus lactis* OTG1204 were selected. After biological assessment including antibiotic and hemolytic activity tests, OTG1204 was selected for its excellence (Supplementary Table S2, Supplementary Figure S1). We further examined the immunostimulatory effects of OTG1204 on RAW 264.7 macrophages. OTG1204 increased NO production in RAW 264.7 macrophages in a concentration-dependent manner without cellular cytotoxicity (Figure 2A) and Polymyxin B (LPS-neutralizing agent) did not influence OTG1204-induced NO production, indicating the absence of endotoxin contamination (Figure 2B). Similar to its effects on NO production, OTG1204 increased PGE_2_ production in a dose-dependent manner (Figure 2C). OTG1204 significantly upregulated the protein and mRNA expression of iNOS and COX-2 in RAW 264.7 macrophages (Figure 2D–F). To investigate the effects of OTG1204 on the production and mRNA expression of cytokines, we performed ELISA and qRT-PCR analyses. As shown in Figure 2G–L, OTG1204 treatment led to a significant elevation in the production and mRNA expression of IL-1β, IL-6, and TNF-α in RAW 264.7 macrophages. 

### 3.2. OTG1204 Activates NF-κB, AP-1, and MAPK Signaling Pathway in RAW 264.7 Macrophages

**Figure 3:**
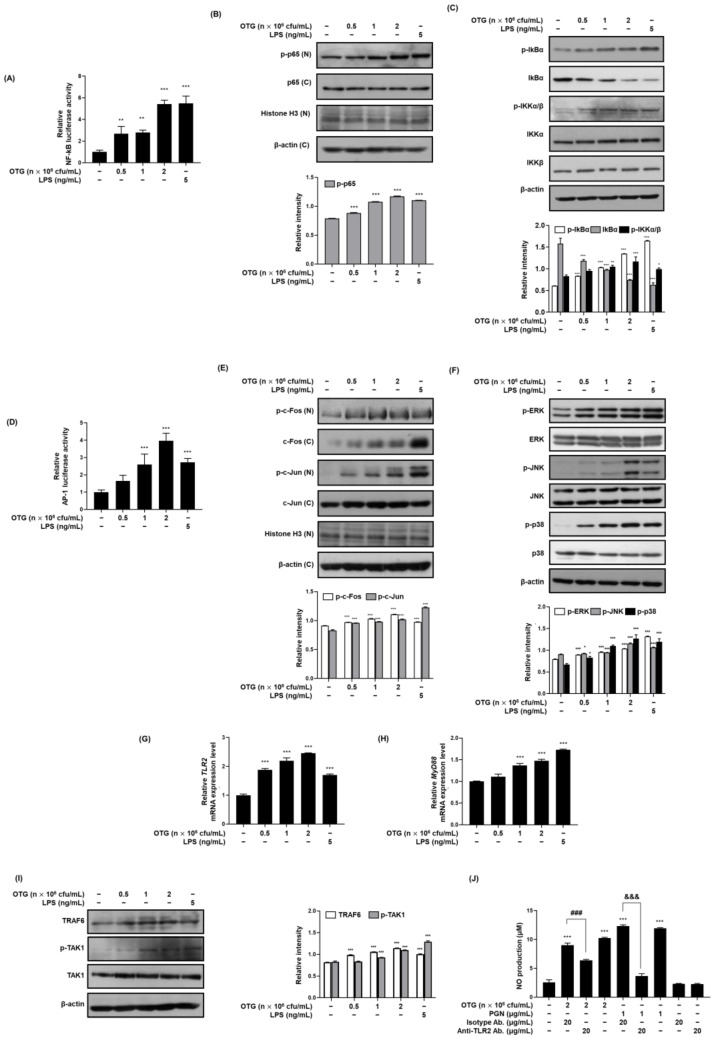
Effects of OTG1204 on NF-κB, AP-1, MAPK, and TLR2 signaling pathways in RAW 264.7 macrophages. (**A**) NF-κB activity was determined by luciferase assay as described in the Section 2. (**B**,**C**) Cells were treated with OTG1204 (0.5 × 10^6^, 1 × 10^6^, or 2 × 10^6^ cfu/mL) for 1 h (p-p65, p65), 30 min (p-IkBα, IkBα) or 15 min (p-IKKα/β, IKKα, IKKβ), and expression levels of proteins were detected by Western blot analysis. LPS (5 ng/mL) was used as the positive control. (**D**) AP-1 activity was determined by luciferase assay as described in the Section 2. (**E**,**F**) Cells were treated with OTG1204 (0.5 × 10^6^, 1 × 10^6^, or 2 × 10^6^ cfu/mL) for 1 h (AP-1) or 20 min (MAPKs), and expression levels of proteins were detected by Western blot analysis. (**G**,**H**) Cells were treated with OTG1204 (0.5 × 10^6^, 1 × 10^6^, or 2 × 10^6^ cfu/mL) for 1 h, the mRNA expressions of TLR2 and Myd88 were determined by qRT-PCR. LPS (5 ng/mL) was used as the positive control. (**I**) Cells were treated with OTG1204 for 2 h (TRAF6) or 30 min (p-TAK1 and TAK1), and then the expression levels of proteins were detected by Western blot analysis. β-actin was used as the internal control. (**J**) After pretreatment with isotype antibody or anti-TLR2 antibody (20 μg/mL) for 1 h, cells were exposed to peptidoglycan (PGN) (1 μg/mL) or OTG1204 (2 × 10^6^ cfu/mL) for 24 h. NO production was measured by Griess reaction, as described in the Section 2. β-actin and Histone H3 were used as the internal control. N—nuclear fraction; C—cytosolic fraction. Data were expressed as means ± SD. * *p* < 0.05, ** *p* < 0.01, *** *p* < 0.001 vs. Control; ^###^ *p* < 0.001 vs. anti-TLR2 Ab treatment; ^&&&^ *p* < 0.001 vs. PGN and anti-TLR2 Ab treatment.

We examined the effect of OTG1204 on transcription factor activity by transfecting cells with a luciferase reporter plasmid. As shown in Figure 3A, reporter gene analysis using pNF-κB-Luc demonstrated that OTG1204 stimulated NF-κB activity in RAW 264.7 macrophages in a concentration-dependent manner. Western blot analysis revealed that OTG1204 significantly increased nuclear phosphorylated-p65, indicating activation of the NF-κB pathway (Figure 3B). Consistently, OTG1204 induced phosphorylation of IKKs and IκBα followed by IκBα degradation in RAW 264.7 macrophages (Figure 3C), whereas the levels of IKKα and IKKβ remained unaffected. In addition, OTG1204 in a dose-dependent manner enhanced AP-1-dependent luciferase activity according to the results of reporter gene analysis (Figure 3D) and stimulated nuclear phosphorylation of c-Fos and c-Jun, which are subunits of AP-1 in RAW 264.7 macrophages (Figure 3E). As shown in Figure 3F, OTG1204 activated ERK, JNK, and p38 in a concentration-dependent manner; however, did not affect the levels of non-phosphorylated ERK, JNK, and p38.

### 3.3. OTG1204 Upregulates TLR2 Signaling Pathway Involving MyD88, TRAF6, and TAK1 in RAW 264.7 Macrophages

TLR2 identifies and responds to initial threats from bacterial infections, subsequently activating downstream immune response which can positively influence the host’s immune response [22]. Therefore, we focused on TLR2-mediated signaling activation in OTG1204-treated RAW 264.7 macrophages. OTG1204 treatment significantly upregulated the mRNA expression of TLR2 and MyD88 and the protein expression of TRAF6 and p-TAK1 in RAW 264.7 macrophages, indicating the engagement of TLR2 signaling pathways (Figure 3G–I). To confirm that TLR2 is involved in OTG1204-induced immunostimulatory effects, we assessed NO production using an anti-TLR2 antibody in OTG1204-treated RAW 264.7 macrophages. As shown in Figure 3J, OTG1204 treatment with or without an isotype control antibody, similar to peptidoglycan (a recognized TLR2 agonist), significantly increased NO production. However, this increase was significantly reduced when an anti-TLR2 antibody was used with OTG1204 or peptidoglycan, as the antibody blocks TLR2 and prevents the downstream signaling required for NO production. These results strongly suggest that OTG1204’s immunostimulatory effects in RAW 264.7 macrophages are mediated through the TLR2 pathway.

### 3.4. OTG1204 Treatment Recovers the Immune State in CTX-Induced Immunosuppression Mice

**Figure 4:**
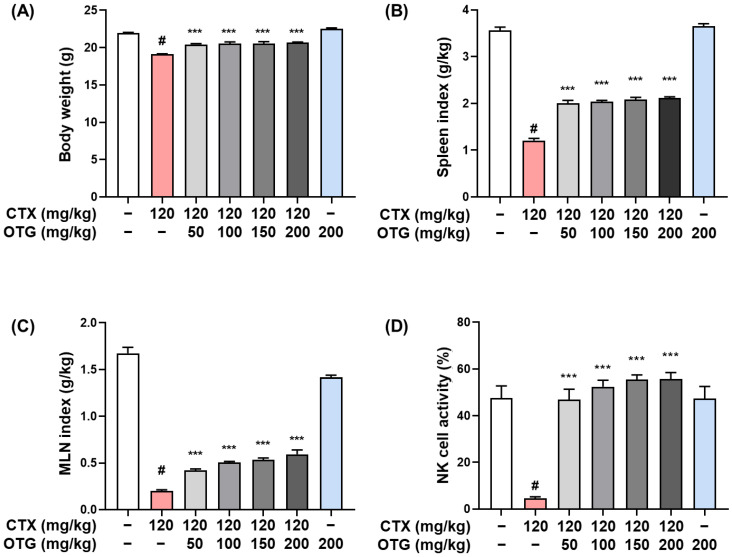
Effects of OTG1204 on body weight, immune organ indices, and NK cell activity in cyclophosphamide (CTX)-induced mice. (**A**) The mice body weights and indices of (**B**) spleen and (**C**) MLN were estimated at the end of the experiments. (**D**) NK cell activity was determined by LDH assay, as described in the Section 2. Data are presented as the mean ± SEM. ^#^ *p* < 0.05 vs. CON group; *** *p* < 0.001 vs. CTX group.

To assess whether OTG1204 exerts immunostimulatory effects in vivo, we evaluated the body weight and immune organ indices in CTX-induced immunosuppressed mice. CTX treatment (120 mg/kg/day, i.p.) potently decreased the body weight compared with those in the CON group (Figure 4A). Treatment with OTG1204 at various doses (50, 100, 150, or 200 mg/kg/day, p.o.) significantly restored the body weight. Next, to evaluate the functional status of immune organs, immune organ indices, including those of the spleen and MLN, were measured. CTX treatment markedly reduced the spleen and MLN indices, but OTG1204 restored these indices in a dose-dependent manner (Figure 4B,C). Additionally, CTX treatment significantly suppressed NK cell activity compared to that in the CON group, indicating that CTX has immunosuppressive effects (Figure 4D). However, the administration of OTG1204 significantly prevented the CTX-induced decline in NK cell activity, demonstrating the potential immunoregulatory role of OTG1204 in enhancing NK cell function.

### 3.5. OTG1204 Treatment Regulates the Proportion of Immune Cells in CTX-Induced Immunosuppression Mice

**Figure 5:**
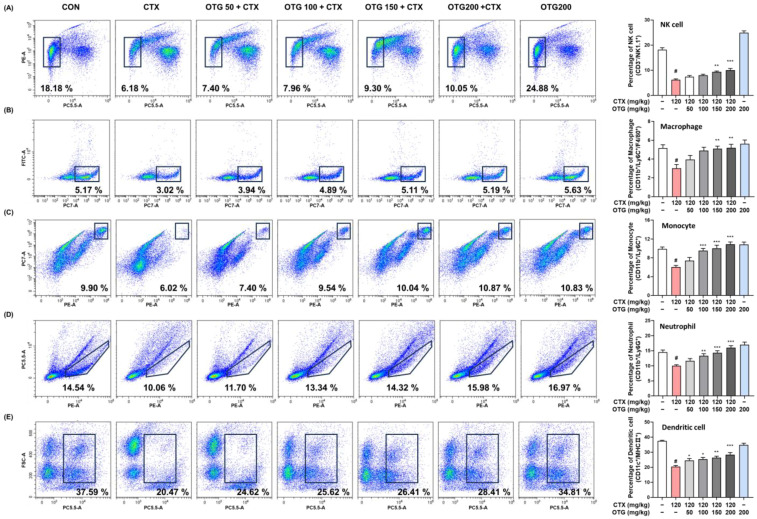
Effects of OTG1204 on innate immune cells in splenocytes of CTX-induced mice. To identify innate immune cells, the isolated splenocytes were stained with fluorescent antibodies indicating marker proteins, as follows: (**A**) CD3^−^/NK1.1^+^; NK cell; (**B**) CD11b^+^/Ly6C^+^/F4/80^+^; macrophage; (**C**) CD11b^+^/Ly6C^+^; monocyte; (**D**) CD11b^+^/Ly6G^+^; neutrophil; (**E**) CD11C^+^/MHCII^+^; dendritic cell and immune cell populations were analyzed by flow cytometry. Data are presented as the mean ± SEM. ^#^ *p* < 0.05 vs. CON group; * *p* < 0.05, ** *p* < 0.01, *** *p* < 0.001 vs. CTX group.

**Figure 6:**
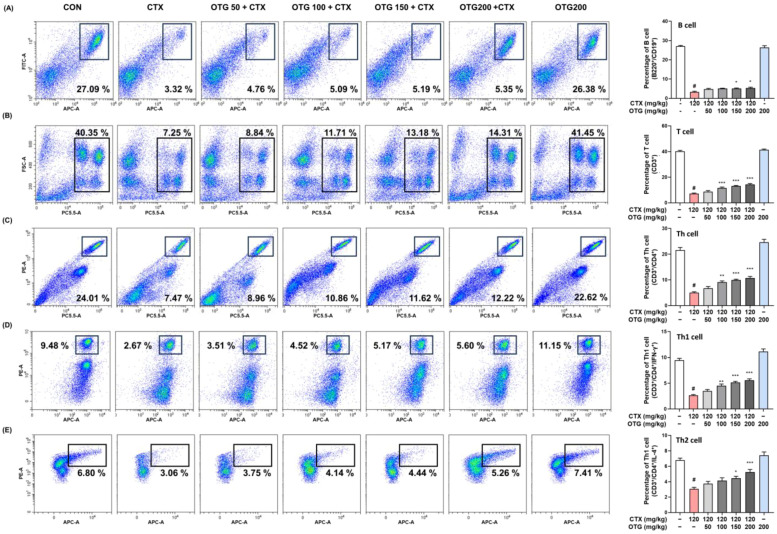
Effects of OTG1204 on adaptive immune cells in splenocytes of CTX-induced mice. To identify adaptive immune cells, the isolated splenocytes were stained with fluorescent antibodies indicating marker proteins, as follows: (**A**) B220^+^/CD19^+^; B cell; (**B**) CD3^+^; T cell; (**C**) CD3^+^/CD4^+^; Th cell; (**D**) CD3^+^/CD4^+^/IFN-γ^+^; Th1 cell; (**E**) CD3^+^/CD4^+^/IL-4^+^; Th2 cell and immune cell populations were analyzed by flow cytometry. Data are presented as the mean ± SEM. ^#^ *p* < 0.05 vs. CON group; * *p* < 0.05, ** *p* < 0.01, *** *p* < 0.001 vs. CTX group.

To investigate the impact of OTG1204 on innate and adaptive immune responses, we determined the proportion of immune cells in the splenocytes of CTX-treated mice. CTX injections notably reduced the percentage of immune cells associated with the innate immune system, such as NK cells, macrophages, monocytes, neutrophils, and dendritic cells (Figure 5A–E). However, OTG1204 treatment significantly recovered these cells to the levels in the CON group in a dose-dependent manner. As shown in Figure 6A–E, CTX markedly suppressed the proportion of cells involved in adaptive immune responses, including B, T, Th, Th1, and Th2 cells; meanwhile, the OTG1204 treatment group regained these adaptive immune cells.

### 3.6. OTG1204 Enhances T Cell-Mediated Cytokines in the Spleen of CTX-Induced Immunosuppression Mice

**Figure 7:**
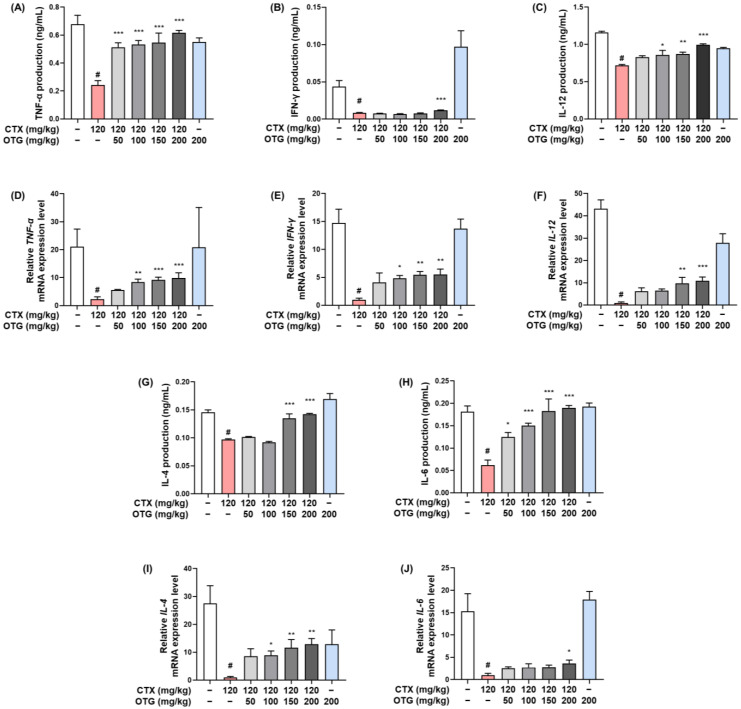
Effects of OTG1204 on the production and mRNA expression of cytokines in splenocytes of CTX-induced mice. For T cell activation, isolated splenocytes were stimulated with ConA (5 μg/mL) for 48 h. Isolated splenocytes were stimulated with ConA (5 μg/mL) for 48 h. (**A**–**C**,**G**,**H**) the cytokine production and (**D**–**F**,**I**,**J**) mRNA expression were measured by ELISA kits and qRT-PCR, respectively. Data are presented as the mean ± SEM. ^#^ *p* < 0.05 vs. CON group; * *p* < 0.05, ** *p* < 0.01, *** *p* < 0.001 vs. CTX group.

To confirm the significant recovery of immune cells by OTG1204, we measured Th1- and Th2-related cytokine levels during ConA-induced T cell proliferation in splenocytes. As shown in Figure 7A–F, CTX injections significantly reduced both the production and mRNA expression of Th1-related cytokines, such as TNF-α, IFN-γ, and IL-12, compared with those in the spleen of the CON group, whereas OTG1204 administration restored these decreases. In addition, OTG1204 treatment upregulated the production and mRNA expression of Th2-related cytokines, including IL-4 and IL-6, compared with those in the CTX group (Figure 7G–J). These results suggest that OTG1204-activated Th cells contribute to immune enhancement in CTX-treated mice. 

### 3.7. OTG1204 Prevents Gut Barrier Disruption in CTX-Induced Immunosuppression Mice

**Figure 8:**
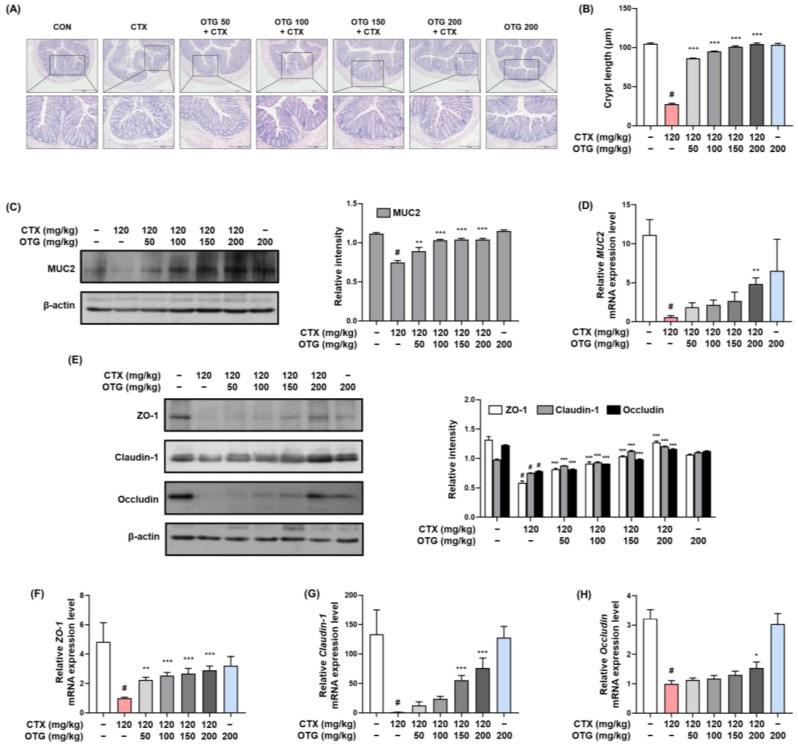
Effect of OTG1204 on gut barrier disruption in CTX-induced mice. (**A**) H&E staining of colon tissue; scale bars, 500 μm and 200 μm. (**B**) Crypt length. (**C**) The protein and (**D**) mRNA expressions of MUC2 were detected by Western blot analysis and qRT-PCR, respectively. (**E**) The protein and (**F**–**H**) mRNA expressions of tight junction-related markers were detected by Western blot analysis and qRT-PCR, respectively. Data are presented as the mean ± SEM. ^#^ *p* < 0.05 vs. CON group; * *p* < 0.05, ** *p* < 0.01, *** *p* < 0.001 vs. CTX group.

We examined the potential protective effects of OTG1204 against CTX-induced intestinal damage by H&E staining analysis. The CON group displayed an integrated and tightly linked morphology with elongated crypts in the colon tissue (Figure 8A,B). Although the CTX group exhibited an irregular morphology and a shortened crypt length, OTG1204 treatment restored histological damage to a level similar to that in the CON group. Additionally, OTG1204 treatment upregulated the mRNA and protein expression of MUC2 in the colons of CTX-induced immunosuppression mice (Figure 8C,D). Our Western blotting and qRT-PCR analyses showed that OTG1204 treatment significantly restored CTX-downregulated ZO-1, claudin-1, and occludin protein and mRNA expression in a dose-dependent manner (Figure 8E–H).

### 3.8. OTG1204 Regulates Gut Microbiota Composition in CTX-Induced Immunosuppression Mice

**Figure 9:**
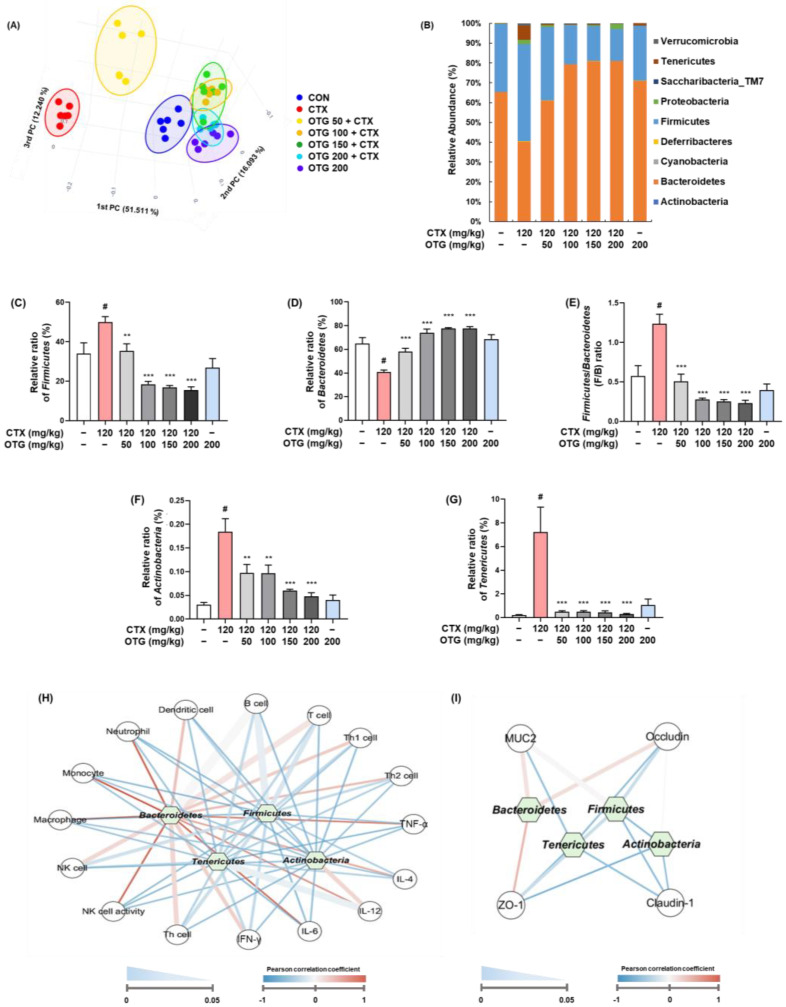
Effect of OTG1204 on gut microbiota composition in CTX-induced mice. (**A**) β-diversity was presented by principal coordinate analysis (PCoA). (**B**) Bacterial taxonomic profiling at the phylum level. Abundance of (**C**) *Firmicutes* and (**D**) *Bacteroidetes* at the phylum level. (**E**) Ratio of *Firmicutes/Bacteroidetes*. Abundance of (**F**) *Actinobacteria* and (**G**) *Tenericutes* at the phylum level. Network analyses of interactions between (**H**) the gut microbiota and various immune cells and cytokines, and (**I**) the gut microbiota and tight junction proteins, assessed by the Spearman coefficient test. Data are presented as the mean ± SEM. ^#^ *p* < 0.05 vs. CON group; ** *p* < 0.01, *** *p* < 0.001 vs. CTX group.

To investigate the impact of OTG1204 on microbiome diversity and composition, we analyzed a PCoA on the β-diversity of different experimental groups after the microbiome taxonomic profiling. As shown in Figure 9A, the CTX group was separated from the CON group in the microbiome cluster, whereas treatment with OTG1204 effectively shifted to the CON group, suggesting that OTG1204 preserves the microbial diversity and composition. At the phylum level, the taxonomic composition of microbial communities, including *Bacteroidetes*, *Firmicutes*, *Verrucomicrobia*, *Tenericutes*, *Saccharibacteria_TM7*, *Proteobacteria*, *Deferribacteres*, *Cyanobacteria*, and *Actinobacteria*, was analyzed; *Bacteroidetes* and *Firmicutes* collectively constituted more than 90 percent of the microbiota (Figure 9B). CTX treatment increased the abundance of *Firmicutes* compared to the CON group, whereas it decreased the abundance of *Bacteroidetes* (Figure 9C,D). However, OTG1204 treatment restored the *Firmicutes* and *Bacteroidetes* abundance altered by CTX treatment. Concomitant with these results, OTG1204 treatment recovered the elevated *Firmicutes*/*Bacteroidetes* ratio in the CTX group (Figure 9E). Furthermore, CTX treatment significantly increased the abundance of *Actinobacteria* and *Tenericutes* compared with that in the CON group, whereas OTG1204 treatment downregulated these microbial populations (Figure 9F,G). The network analyses revealed significant interactions between the gut microbiota and immune cells, cytokines, and tight junction proteins. (Figure 9H,I). *Bacteroidetes* exhibited strong positive correlations (red lines) with NK cell activity, monocyte, and neutrophil populations, as well as cytokines such as IL-4 and IL-6. Conversely, *Firmicutes* negatively correlated (blue lines) with NK cell activity, monocyte population, and cytokines including IL-6, showing an opposite trend to *Bacteroidetes*. *Tenericutes* and *Actinobacteria* also displayed negative relationships with immune markers such as TNF-α, IL-4, and Th cell population. Additionally, *Bacteroidetes* were positively linked with ZO-1, while *Firmicutes* showed strong negative correlations with claudin-1. *Tenericutes* and *Actinobacteria* had negative links to claudin-1 and ZO-1, respectively. These results underscore the complex regulatory roles of gut microbiota in immune modulation and intestinal barrier function. 

## 4. Discussion

As the immune system serves as the body’s defense mechanism, imbalances in the immune response can increase susceptibility to various diseases, including type 1 diabetes, allergies, cancer, and rheumatoid arthritis [23,24]. Consequently, research into the development of chemical synthesis-based immunostimulants that can enhance or suppress the immune system has been carried out. However, these agents carry risks of side effects, such as toxicity, drowsiness, fatigue, constipation, low blood cell counts, and neuropathy [25]. Therefore, the development of natural immunostimulants, such as probiotics, which have minimal side effects, has emerged as a crucial and promising strategy for safer and more natural immune regulation and disease risk reduction [26]. Following the growing need for safer immune modulators, we screened 20 strains of LAB isolated from traditional Korean fermented foods to identify potential candidates with immunostimulatory activity. Based on evaluations of cell viability, NO production, antibiotic resistance, and hemolytic activity, OTG1204 was selected as a promising novel probiotic strain with immune-enhancing potential. This strain demonstrated strong safety and efficacy profiles, making it a suitable candidate for further development in natural immunostimulant research. *L. lactis* LM1185, a strain of *L. lactis*, induces the secretion and expression of immune mediators such as NO and cytokines [27]. In the present study, OTG1204 promoted NO and PGE_2_ production and upregulated the protein and mRNA expression of iNOS and COX-2 in macrophages. Additionally, polymyxin B, a specific LPS-induced TLR4 activation inhibitor, did not affect OTG1204-induced NO production, which is considered OTG1204-induced activation caused by non-endotoxin contamination. These findings reveal the potential of OTG1204 as a safe and effective immunostimulatory agent that can enhance immune responses without adverse effects. Furthermore, OTG1204 did not cause hepatotoxicity (GOT/GPT) or nephrotoxicity (BUN and creatinine) (Supplementary Figure S2).

Cytokine production by activated macrophages orchestrates the immune response by recruiting and activating T lymphocytes and other immune cells [28]. We observed that OTG1204 stimulated both the production and mRNA expression of IL-1β, IL-6, and TNF-α, suggesting that OTG1204 activates macrophages by increasing the secretion and expression of immune mediators, including NO, PGE_2_, and cytokines. Although various signaling pathways influence immune responses, two key signaling molecules, NF-κB and MAPK/AP-1, are vital for the functioning of the immune system [29]. These pathways control both innate and adaptive immunity by regulating the expression of numerous cytokines, transcription factors, and regulatory proteins [30]. Probiotics such as the heat-killed *Weissella cibaria* JW15 increased NF-κB activation in RAW 264.7 macrophages, demonstrating their role in modulating the immune response [31]. Similarly, the immunostimulatory effects of *L. lactis* subsp. *lactis* CAB701 triggered the phosphorylation of JNK, indicating that this probiotic activates the MAPK pathway to modulate immune responses [32]. Although OTG1204 activated the NF-κB and MAPK/AP-1 signaling pathways, its upstream pathway involving TLR2, which is one of the key receptors for bioactive probiotics and a mediator of several signaling pathways, remained unclear. Upon TLR2 activation, the recruitment of Myd88 leads to an interaction with TRAF6, which influences downstream factors such as the TAK1, MAPK, and NF-κB signaling pathways [33]. Lee et al. found that *Lactobacillus plantarum* HY7712 exerts immunomodulating effects by activating the TLR2/NF-κB signaling pathway in RAW 264.7 cells [34]. In the present study, OTG1204 enhanced TLR2 and MyD88 mRNA expression and upregulated TRAF6 and phosphorylated TAK1 protein levels. Based on these results, the immune-enhancing mechanism of OTG1204 may involve the activation of NF-κB and MAPK/AP-1 via TLR2 activation followed by enhanced secretion of immune mediators, such as NO, PGE_2_, IL-1β, IL-6, and TNF-α, in RAW 264.7 macrophages.

To verify these in vitro results, we evaluated the immunostimulatory effects of OTG1204 in a murine model of CTX-induced immunosuppression. Although CTX is commonly used in chemotherapy for tumors or autoimmune diseases, it induces immunosuppression primarily through its cytotoxic effects on rapidly dividing cells, including immune cells [35]. CTX functions by causing DNA cross-linking and strand breaks, which lead to cell cycle arrest and trigger apoptosis or necrosis in immune cells, particularly lymphocytes. This depletion of immune cells, especially T and B cells, weakens both innate and adaptive immune responses, leaving the body more vulnerable to infections and immune dysfunction. Additionally, CTX is known to disrupt the gut microbiota, causing dysbiosis [36], making this model used for assessing both the immune-enhancing effects of OTG1204 and its potential to regulate the microbiota. Thus, many studies have established CTX-induced immunosuppressive animal models and provided a foundation for testing the protective or restorative effects of immunostimulatory agents [20,21]. OTG1204 treatment recovered the CTX-reduced body weight as well as indices of spleen and MLN, which promote the activation and maturation of immune cells, thereby enhancing the body’s defense mechanisms against infections and diseases [37]. Additionally, OTG1204 significantly restored immunoglobulin G (IgG) production in the serum of CTX-induced mice (Supplementary Figure S3). Increased IgG levels indicate an enhanced humoral immune response, reflecting improved overall immune function [38]. To respond to pathogens and injury, innate immune cells, such as neutrophils, macrophages, monocytes, and NK cells, have evolved mechanisms to detect microbes and fight infections [39]. As NK cells are well-known sentinel cells of the innate immune system that can recognize and kill infected cells, NK cell activity is used as a marker of general immunological functions [40]. Accordingly, we determined the NK cell activity in splenocytes from CTX-treated mice and observed that OTG1204 significantly enhanced NK cell activity in a dose-dependent manner. Monocytes, as part of the innate immune system, can differentiate into macrophages and monocyte-derived dendritic cells [41]. Macrophages and neutrophils play pivotal roles in phagocytosis and presenting antigens to T cells, thereby facilitating the bridge between innate and adaptive immunity [42]. Similarly, dendritic cells act as messengers between the innate and adaptive immune systems and activate T cells [43]. Within the adaptive immune system, B cells are responsible for humoral immunity by producing immunoglobulins and antibodies that neutralize foreign antigens [44]. In particular, T cells, which are produced in the bone marrow and mature in the thymus, transform into Th cells upon encountering antigens, which then activate other immune cells and work with B cells to produce antibodies [45] Therefore, we determined the effects of OTG1204 on the population of innate and adaptive immune cells in the spleen of CTX-treated mice. The CTX group showed a reduced percentage of innate-related immune cells, including NK cells, macrophages, monocytes, neutrophils, and dendritic cells, and adaptive immune-related cells, such as B, T, Th, Th1, and Th2 cells. However, OTG1204 treatment significantly restored the population of immune cells in CTX-treated mice. Among these immune cells, OTG1204 more significantly upregulated T cells than it did B cells, indicating that OTG1204 has a specific preventive effect on T cells. Th cells are classified as Th1 and Th2; OTG1204 treatment not only restored the generation of Th1-related cytokines (TNF-α, IFN-γ, and IL-12) but also upregulated Th2-related cytokines (IL-4 and IL-6) in ConA-activated splenocytes. Based on these findings, OTG1204 significantly restores immune cell populations and enhances both innate and adaptive immune responses in CTX-induced immunosuppressed mice, effectively mitigating immunosuppression and improving overall immune function.

The large intestine plays a crucial role in maintaining immune homeostasis by serving as the primary site for probiotic colonization and interaction with immune cells, which strengthens the gut barrier [46]. Additionally, the gut hosts a complex microbial community that is essential for mucosal immunity and maintaining intestinal balance during external environmental changes [47]. This balance is reinforced by gel-forming mucin and tight junctions, which ensure the integrity of the epithelial and proper mucosal barrier function in the gut [48]. In the present study, treatment with OTG1204 restored the mRNA and protein expression of MUC2 and tight junction-related proteins (occludin, claudin-2, and ZO-1) compared with those in the CTX group. In intestinal epithelial cells, probiotics modulate the host immune response via TLR2, which maintains tight junction proteins and activates NF-κB signaling [49]. For example, *Lactobacillus casei* NCU011054 also improved CTX-induced intestinal immunosuppression by correcting the imbalance of Th1/Th2 and upregulating T-bet/GATA-3 and IFN-γ/IL-4 via the TLR2/NF-κB pathway [50]. Similarly to this report, we also observed that OTG1204 increased not only TLR2 mRNA expression but also phosphorylated p65 expression in the colon tissue (Supplementary Figure S4), indicating that OTG1204 stimulated immune responses through the NF-κB pathway mediated by TLR2 activation in the colon.

The gut microbiota regulates epithelial permeability through tight junction modulation to prevent potentially harmful antigens [51]. Microbiota has been linked to multiple immune functions, including cytokine production, homeostasis maintenance, T cell production, and immune system regulation [52]. The gut microbiota metabolizes dietary components and generates various active microbial metabolites depending on the type of diet. These metabolites exert direct effects on the gut epithelium to regulate barrier function, thereby activating mucosal immunity through various pathways [53]. However, CTX leads to gut dysbiosis, which is characterized by an imbalanced microbiota composition and is associated with autoimmune diseases [54]. Probiotics restore the gut microbiome composition and provide positive effects on gut microbial communities, preventing gut inflammation and other intestinal or systemic disease [55]. Previous studies demonstrated that oral administration of three complex probiotics, *Bifidobacterium animalis* subsp. *lactis* XLTG11, *Lacticaseibacillus casei* Zhang, and *Lactiplantibacillus plantarum* P8, reversed the Chao1 index decreased by CTX treatment and separated to the CTX group in β-diversity analysis of the microbial composition [56]. In agreement with these findings, our data showed that the OTG1204 group had a gut microbiota composition closer to that of the CON group than to that of the CTX group. Furthermore, for the taxonomic composition at the phylum level, OTG1204 reduced the abundance of *Firmicutes* and increased *Bacteroidetes* which had been altered by CTX injection. As *Bacteroidetes* and *Firmicutes* constitute more than 90% of the microbiota, the balance between *Bacteroidetes* and *Firmicutes* is important in homeostasis maintenance [57]. *Actinobacteria* are involved in ecological processes, including the production of vitamins and the synthesis of antibiotics [58]. However, some members of the *Actinobacteria* such as *Corynebacterium* and *Mycobacterium* can be pathogenic to humans [59]. *Tenericutes* are found in animals and plants, and some species within this group, including *Mycoplasma*, cause infections such as pneumonia and urinary tract infections in animals and humans [60]. In the present study, OTG1204 treatment decreased the CTX-induced abundance of *Actinobacteria* and *Tenericutes*, which are notable pathogens in various contexts. Although these pathogens constituted a relatively small proportion of the microbiome, further studies are required to provide more detailed information regarding their roles. Additionally, we analyzed the correlations between the changes in four phylum-level bacteria influenced by OTG1204 (*Bacteroidetes*, *Firmicutes*, *Tenericutes*, and *Actinobacteria*) and immune cells, cytokines secreted by immune cells, and tight junction proteins. *Bacteroidetes*, known for maintaining immune homeostasis and promoting anti-inflammatory responses, showed positive correlations with immune-modulating factors. Conversely, *Firmicutes*, which exhibited negative correlations, include several harmful bacterial species associated with pro-inflammatory effects and disruption of the gut barrier [61]. *Tenericutes* and *Actinobacteria* also exhibited negative correlations and are associated with similar harmful effects. These findings highlight the significant impact of OTG1204 on gut microbiota composition and its subsequent effects on immune modulation and intestinal barrier function. By promoting a healthier balance of beneficial bacteria and reducing harmful bacteria, OTG1204 mitigates CTX-induced dysbiosis and supports overall gut health and immune homeostasis. This underscores its potential as a therapeutic agent for enhancing immune health through the modulation of gut microbiota.

## 5. Conclusions

*Lactococcus lactis* OTG1204 exerted immunostimulatory effects in RAW 264.7 macrophages and CTX-induced immunosuppressive animal models. OTG1204 activated the NF-κB and MAPK/AP-1 pathways via TLR2-mediated immune stimulatory signaling in macrophages. Moreover, oral administration of OTG1204 restored NK cell activity, the populations of both innate and adaptive immune cells, and the cytokines released by Th cells. In addition, OTG1204 prevented the CTX-induced changes in MUC2 and tight junction-related protein and mRNA expression and also altered the microbiota composition. Based on our results, OTG1204 shows potential as a functional probiotic supplement to increase immunostimulatory functions.

## Figures and Tables

**Table 1:** Primer sequences used in qRT-PCR.

Gene	Forward Sequence	Reverse Sequence
*Claudin-1*	ACAGTGCAAAGTCTTCGATT	ATTCTTGCACCTCATCATC
*COX-2*	GGAGAGACTATCAAGATAGT	ATGGTCAGTAGACTTTTACA
*Occludin*	TGGCGGATATACAGACCCAA	CGATCGTGGCAATAAACACC
*IL-4*	TTGTCATCCTGCTCTTCTTT	TCTTCTTCAAGCATGGAGTT
*IL-6*	GAGGATACCACTCCCAACAGACC	AAGTGCATCATCGTTGTTCATACA
*IL-1* *β*	ACCTGCTGGTGTGTGACGTT	TCGTTGCTTGGTTCTCCTTG
*IL-12*	TCTGCAGAGAAGGTCACACT	ATGAAGAAGCTGGTGCTGTA
*iNOS*	AATGGCAACATCAGGTCGGCCATCACT	GCTGTGTGTCACAGAAGTCTCGAACTC
*IFN-* *γ*	GCTGATCCTTTGGACCCTCT	AGAGCTGCAAAGCCAAGATG
*MUC2*	ATGCCCACCTCCTCAAAGAC	GTAGTTTCCGTTGGAACAGTGAA
*TLR2*	GTTGTTCCCTGTGTTGCTGG	GAGTTCGCAGGACCAAACAA
*TNF-α*	AGCACAGAAAGCATGATCCG	CTGATGAGAGGGAGGCCATT
*ZO-1*	CTTCTCTTGCTGGCCCTAAAC	TGGCTTCACTTGAGGTTTCTG
*β-actin*	ATCACTATTGGCAACGAGCG	TCAGCAATGCCTGGGTACAT

## Data Availability

The original contributions presented in the study are included in the article and Supplementary Materials, further inquiries can be directed to the corresponding author.

## Supplementary Materials

The following supporting information can be downloaded at: https://www.mdpi.com/article/10.3390/nu16213629/s1, Table S1: Effect of LAB strains on cell viability and NO production in RAW264.7 cells; Table S2: MIC of the selected strains against nine antibiotics; Table S3: Antibodies used in the Western blot analysis; Figure S1: Test for hemolytic activity. Figure S2: Effects of OTG1204 on the plasma level of GOT, GPT, BUN, and creatinine production in CTX-induced mice; Figure S3: Effects of OTG1204 IgG production in CTX-induced mice; Figure S4: Effects of OTG1204 on the mRNA expression of TLR2 and protein expression of p-p65 in the large intestine of CTX-induced mice.
