# Supplementary material for: Immunostimulation Signaling via Toll-like Receptor 2 Activation: A Molecular Mechanism of Lactococcus lactis OTG1204 In Vitro and In Vivo
Source: Nutrients. 2024 Oct 25;16(21):3629. doi: 10.3390/nu16213629 (PMC11547582; doi:10.3390/nu16213629)
Supplement: Supplementary file 1 [file nutrients-16-03629-s001.zip › nutrients-3254083-supplementary.pdf]

Supplementary Data

Supplementary Materials and Methods

Measurement of antibiotic resistance and hemolytic activity

The minimum inhibitory concentrations (MIC) against nine antibiotics were measured using commercial E-test strips (bioMerieux, Marcy l'Etoile, France). Cultures of each strain were streaked on the MRS agar plates (BD Difco, Sparks, MD, USA) using sterilized cotton swabs. E-test strips were then placed on the surface of the agar plates and incubated at 37℃ for 48 h. The MIC was determined as the lowest value of the strip, which the bacterial growth was inhibited. For hemolytic activity, the cultured strains were streaked on the blood agar plates supplemented with 5% sheep blood (KisanBio, Seoul, Korea). After incubation at 37℃ for 48 h, the clear zones around the colonies were observed.

Measurement of glutamic oxaloacetic transaminase (GOT), glutamic pyruvic transaminase (GPT), blood urea nitrogen (BUN), and creatinine in plasma of CTX-induced mice

Whole blood samples were gathered using tubes coated with heparin, from which plasma was separated for the biochemical analysis, including GOT, GPT, BUN, and creatinine. The procedures for preparing and conducting the biochemical test of plasma were followed by the guidelines of T&P Bio Co. (Gwangju, Republic of Korea).

Measurement of immunoglobulin G (IgG) production in the serum of CTX-induced mice

For the serum preparation, the collected blood was left at room temperature for 30 min and centrifuged at 3000 rpm (25°C, 7 min). The supernatant was moved to a new tube and used in the determination of IgG levels by ELISA kits under protocols provided by Invitrogen.

Supplementary Tables

Supplementary Table S1. Effect of LAB strains on cell viability and NO production in RAW264.7 cells

Genus	Species	Strains	Cell Viability (%)	NO (μM)
Untreated			100.00 ± 0.26	-2.50 ± 0.16
LPS (5 ng/ml)			90.06 ± 0.24	28.15 ± 0.45
Lacticaseibacillus	*casei*	OTG1049	88.35 ± 0.13	21.25 ± 0.01
	paracasei	OTG1009	90.51 ± 0.23	3.54 ± 0.04
		OTG1026	90.36 ± 0.02	21.69 ± 0.03
		OTG1033	90.74 ± 0.01	24.60 ± 0.07
Lactiplantibacillus	*plantarum*	OTG1003	97.32 ± 0.01	21.85 ± 0.08
		OTG1023	88.35 ± 0.13	27.54 ± 0.06
		OTG1036	84.66 ± 0.02	24.80 ± 0.05
		OTG1039	87.00 ± 0.07	28.62 ± 0.03
		OTG1051	81.63 ± 0.02	24.16 ± 0.12
		OTG1055	80.34 ± 0.02	20.48 ± 0.04
		OTG1075	84.79 ± 0.23	28.01 ± 0.11
		OTG1113	95.75 ± 0.02	18.01 ± 0.06
		OTG1151	99.37 ± 0.06	17.01 ± 0.05
		OTG1162	85.70 ± 0.21	2.47 ± 0.11
		OTG1165	99.63 ± 0.09	11.17 ± 0.10
Limosilactobacillus	*fermentum*	OTG1050	94.31 ± 0.12	29.48 ± 0.03
Lactococcus	*lactis*	OTG1204	89.29 ± 0.09	34.90 ± 0.12
Levilactobaillus	*brevis*	OTG1072	93.61 ± 0.08	12.49 ± 0.10
		OTG1133	93.77 ± 0.02	21.32 ± 0.05
Weissella	*cibaria*	OTG1078	87.64 ± 0.06	25.43 ± 0.09

Supplementary Table S2. MIC of the selected strains against nine antibiotics

Antibiotics	*L. plantarum*		*L. fermentum*		*Lc. lactis*	
	Cut-off value [a] (μg/ml)	OTG1039	Cut-off value [a] (μg/ml)	OTG1050	Cut-off value [a] (μg/ml)	OTG1204
Ampicillin	2	0.125/S[2]	2	0.125/S	2	0.25/S
Vancomycin	n.r.[1]	256>/S	n.r.	256>/S	4	0.38/S
Gentamicin	16	16/S	16	4/S	32	8/S
Kanamycin	64	256>/R[3]	32	96/R	64	16/S
Streptomycin	n.r.	64/S	64	48/S	32	32/S
Erythromycin	1	0.25/S	1	0.25/S	1	0.125/S
Clindamycin	2	0.25/S	1	0.023/S	1	0.032/S
Tetracycline	32	8/S	8	2/S	4	0.25/S
Chloramphenicol	8	3/S	4	2/S	8	4/S

[a] EFSA (European Food Safety Authority), 2012; [1]n.r. not required; [2]S susceptible; [3]R resistant.

Supplementary Table S3. Antibodies used in the western blot analysis

Supplier	Location	Antibodies
Santa Cruz Biotechnology	Santa Cruz, CA, USA	iNOS, p65, IkBα, c-Fps, p-c-Jun, p-ERK, ERK, p-JNK, JNK, TRAF6, claudin-1, β-actin
Cell signaling Technology	Danvers, MA, USA	p-p65, p- IkBα, p-IKKα/β, IKKα, IKKβ, p-c-Fos, c-Jun, p-p38, p38, TAK1, occludin, Histone H3
Cayman Chemical	Ann Arbor, MI, USA	COX-2
Invitrogen	CA, USA	p-TAK1, ZO-1, MUC2

Supplementary Figures

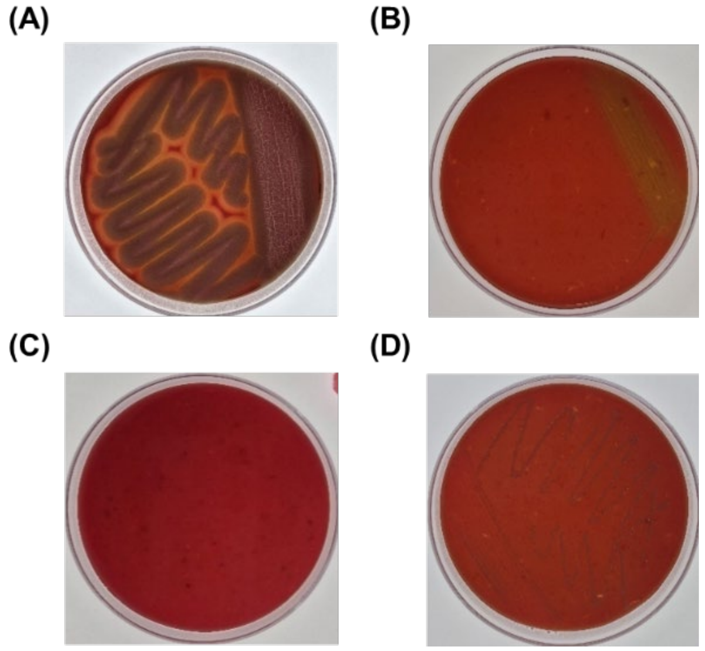

Supplementary Figure S1. Test for hemolytic activity. (A) *B. cereus*; (B) *L. plantarum* OTG1039; (C) *L. fermentum* OTG1050 (D) *Lc. lactis* OTG1204.

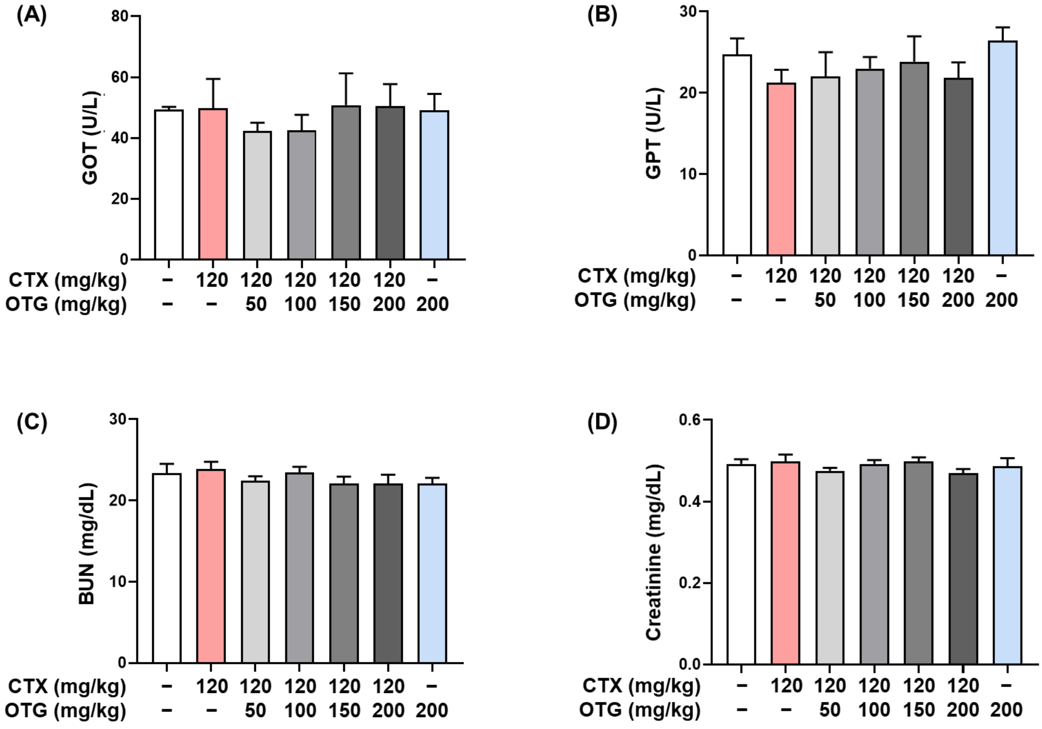

Supplementary Figure S2. Effects of OTG1204 on the plasma level of GOT, GPT, BUN, and creatinine production in CTX-induced mice. The production of (A) GOT, (B) GPT, (C) BUN, and (D) Creatine were

determined by the ELISA kit. Data are presented as the mean ± SEM.

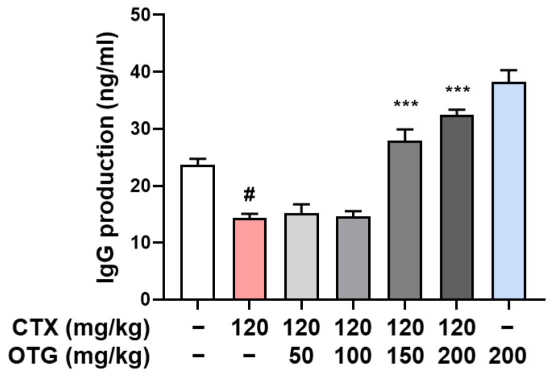

Supplementary Figure S3. Effects of OTG1204 IgG production in CTX-induced mice. The IgG production in serum was detected by ELISA kits. Data are presented as the mean ± SEM. #P < 0.05 vs. CON group; ***P < 0.001 vs. CTX group.

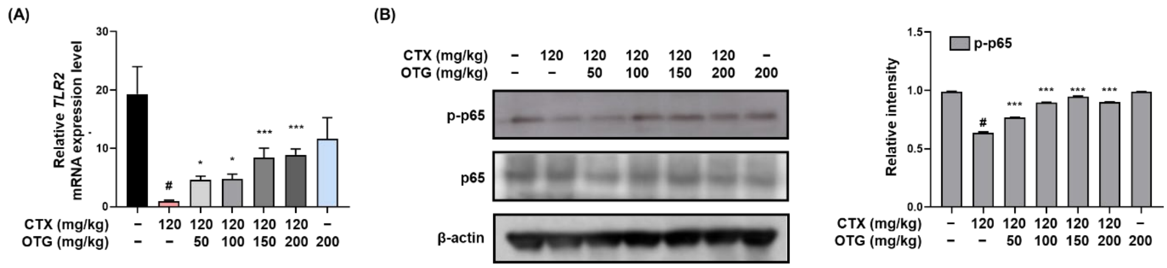

Supplementary Figure S4. Effects of OTG1204 on the mRNA expression of TLR2 and protein expression of p-p65 in the large intestine of CTX-induced mice. (A) The mRNA expression of TLR2 in the large intestine was detected by qRT-PCR analysis. (B) The protein expressions of p-p65 and p65 in the large intestine were detected by western blot analysis. Data are presented as the mean ± SEM. $^{\#}P < 0.05$ vs. CON group; $^{*}P < 0.05$, $^{***}P < 0.001$ vs. CTX group.
